# Exertional heat stroke in a marathon runner with extensive healed deep burns: a case report

**DOI:** 10.1186/1865-1380-4-12

**Published:** 2011-03-22

**Authors:** Puneet Seth, Poh Juliana

**Affiliations:** 1 Department of Emergency medicine, Singapore General Hospital, Singapore; 2 Academy of Medicine, Singapore; 3 Royal College of Surgeons of Edinburgh, Edinburgh, UK

## Abstract

Exertional heat illness typically occurs over hours in younger athletic patients or military recruits who exercise at elevated temperatures for a sufficient period of time to cause the rate of heat production to exceed the capacity of the body to dissipate heat. Since the physiological response to exercise includes cutaneous vasodilation and sweating, any limitation of such a response can cause rapid hyperthermia and thus heat stroke. One such condition is extensive burns healed by cicatrisation of the skin where the scar and grafted skin surface do not have functional sweat glands and are unable to lose heat in response to high temperatures. The authors report one unique case of a female marathon runner with exertional heat stroke who had recovered from deep second and third degree burns over approximately 50% of her body a few years ago.

## Introduction

Exertional heat injuries are known to affect marathoners and army recruits under hot and humid environmental conditions [1,2]. This occurs when heat production exceeds the body's ability to dissipate heat. Since peripheral vasodilation and sweating can dramatically increase heat loss, the lack of these physiological responses seriously predisposes those with these conditions to exertional heat injuries. The authors report a unique case of a female marathon participant who suffered exertional heat stroke possibly caused by her inability to sweat over a large surface area of her body and thus accumulating heat rapidly.

## Case report

A 36-year-old female who had been running a marathon was brought to the emergency department (ED). She was brought in by the Civil Defence ambulance after she had collapsed at the 10 km mark. According to bystander accounts, she was unresponsive, trembling and her eyes were rolling up. There was no jerking of the limbs to suggest a generalised seizure according to the paramedics.

The patient's sister, who was running ahead of her, said that the patient had been well before the marathon and that both had flown in from Australia for the event. The patient used to run regularly, but shorter distances.

On arrival, the patient was noted to be obviously confused and disorientated, and kept trying to get off the bed. Her rectal temperature was 41.6°C initially and dropped to 38.4°C at the emergency department. The heart rate was 120 beats/min, blood pressure was 91/48 mmHg, and the oxygen saturation was 98% on room air. Normal saline was administered through an iv cannula in the left antecubital fossa. The cardiovascular and abdominal examination was unremarkable. She was able to move all four limbs, and her pupils were equal and reactive to light.

**Figure 1:**
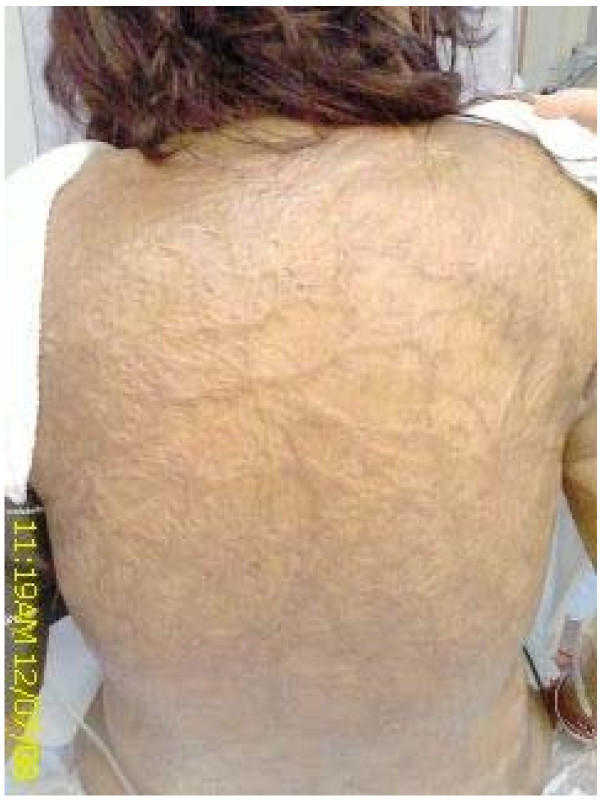
**Skin on the patient's back**.

She was noted to have extensive scarring all over her trunk and upper limbs except the hands and the upper part of her face. Previous case records showed that she had sustained deep second and third degree burns over 49% of her body 5 years earlier. This condition was complicated by the development of a deep vein thrombosis of the right lower limb. The cicatrised skin was noted to be rubbery, firm, dry and shiny (Figure 1).

Rapid evaporative cooling using wet gauze to moisten the skin and pedestal fans at 22°C was employed. One litre of normal saline was infused via two intravenous lines. The patient became progressively calmer, but stayed amnesic throughout her stay at the ED.

Her renal function, liver function and coagulation profile remained within normal limits at all times. Electrolytes showed the presence of some compensated metabolic acidosis (pH 7.40); pCO_2 _was 28.2 mmHg, pO_2 _98 mmHg and serum bicarbonate 16 mmol/l. Base excess was -7.3 (normal -2 to +2). Her creatinine kinase levels peaked at about 2,096 U/l (range: 38 - 164 U/l) before trending downwards. The urine myoglobin level was noted to be a maximum of 100 UG/l (normal < 21 UG/l).

The patient was admitted to the medical ward and stayed there for 3 days. She regained her normal mental state by the evening of the same day. She was eventually discharged with advice to refrain from participating in any such endurance events because of her singular physiology.

## Discussion

Heatstroke is traditionally divided into exertional and classic varieties [3,4], which are defined by the underlying aetiology, but are clinically indistinguishable. Exertional heat illness typically occurs over hours in younger athletic patients or military recruits who exercise at elevated temperatures for a sufficient period of time to cause the rate of heat production to exceed the capacity of the body to dissipate heat. Since the physiological response to exercise includes vasodilation and sweating, any limitation of such a response can cause rapid hyperthermia and thus heat stroke.

The body's ability to dissipate heat by perspiration can be overwhelmed in subjects with normal physiology under extreme conditions. The role of adequate and appropriate rehydration before and during exercise has always been emphasised. This is because it is presumed that the increased heat production and the resultant increase in the core body temperature will drive the peripheral vasodilation and that the sweat secretion rate will increase proportionately to enhance heat loss. This has been established in numerous studies [4,5]. However, it is also known that this proportionate increase in the heat-releasing compensatory mechanism is limited to a certain level beyond which it is overwhelmed and the patient develops hyperthermia.

The present case is unique. The patient had sustained deep second and third degree burns over 49% of her body 5 years earlier, which had healed with the formation of a cicatrix. While some sweat glands may survive superficial second degree burns, most are destroyed or rendered nonfunctional in deep second degree burns^7 ^Additionally, the patient went through multiple partial thickness skin grafting procedures, and such grafts are known to have no sweat glands.

Thus, the patient was left with only about half of her body surface area able to dissipate heat by perspiration and vasodilation. This was probably not enough to maintain normothermia during her marathon endeavour.

Some investigators have pointed out that under moderate conditions of heat, the remaining normal skin can compensate by increased sweating [6,7]. The exact percentage of normal skin required is not known, but is inferred to be in the range of 50-70% based on some studies [8,9,7]. Roskind et al. found a dramatic diminution in heat tolerance in patients with healed burns covering more than 40% of their body surface area [10].

While there is definite scope for further studies in this area, it is perhaps safe to conclude that persons with deep burns to more than 30-40% of their body surface area should be advised against participation in any endurance sports or working in high ambient temperature conditions to avoid heat injury.

## Competing interests

The authors declare that they have no competing interests.

## Authors' contributions

PS compiled the records and initial draft of the report. JP helped with the discussion and editing the manuscript.
